# Transcriptomic signatures reveal a shift towards an anti-inflammatory gene expression profile but also the induction of type I and type II interferon signaling networks through aryl hydrocarbon receptor activation in murine macrophages

**DOI:** 10.3389/fimmu.2023.1156493

**Published:** 2023-05-23

**Authors:** Johannes R. Schmidt, Janine Haupt, Sina Riemschneider, Christoph Kämpf, Dennis Löffler, Conny Blumert, Kristin Reiche, Ulrike Koehl, Stefan Kalkhof, Jörg Lehmann

**Affiliations:** ^1^ Department of Preclinical Development and Validation, Fraunhofer Institute for Cell Therapy and Immunology, Leipzig, Germany; ^2^ Fraunhofer Cluster of Excellence Immune-Mediated Diseases (CIMD), Leipzig, Germany; ^3^ Department of Diagnostics, Fraunhofer Institute for Cell Therapy and Immunology, Leipzig, Germany; ^4^ Institute for Clinical Immunology, Medical Faculty, Leipzig University, Leipzig, Germany; ^5^ Department of Applied Sciences, Institute for Bioanalysis, Coburg University of Applied Sciences and Arts, Coburg, Germany

**Keywords:** aryl hydrocarbon receptor, macrophage activation, innate immunity, immunomodulation, transcriptomics, benzo[*a*]pyrene, indol-3-carbinol, type I/II interferons

## Abstract

**Introduction:**

The aryl hydrocarbon receptor (AhR) is a ligand-activated transcription factor that regulates a broad range of target genes involved in the xenobiotic response, cell cycle control and circadian rhythm. AhR is constitutively expressed in macrophages (Mϕ), acting as key regulator of cytokine production. While proinflammatory cytokines, i.e., IL-1β, IL-6, IL-12, are suppressed through AhR activation, anti-inflammatory IL-10 is induced. However, the underlying mechanisms of those effects and the importance of the specific ligand structure are not yet completely understood.

**Methods:**

Therefore, we have compared the global gene expression pattern in activated murine bone marrow-derived macrophages (BMMs) subsequently to exposure with either benzo[*a*]pyrene (BaP) or indole-3-carbinol (I3C), representing high-affinity vs. low-affinity AhR ligands, respectively, by means of mRNA sequencing. AhR dependency of observed effects was proved using BMMs from AhR-knockout (*Ahr^-/-^*) mice.

**Results and discussion:**

In total, more than 1,000 differentially expressed genes (DEGs) could be mapped, covering a plethora of AhR-modulated effects on basal cellular processes, i.e., transcription and translation, but also immune functions, i.e., antigen presentation, cytokine production, and phagocytosis. Among DEGs were genes that are already known to be regulated by AhR, i.e., *Irf1*, *Ido2*, and *Cd84*. However, we identified DEGs not yet described to be AhR-regulated in Mϕ so far, i.e., *Slpi*, *Il12rb1*, and *Il21r.* All six genes likely contribute to shifting the Mϕ phenotype from proinflammatory to anti-inflammatory. The majority of DEGs induced through BaP were not affected through I3C exposure, probably due to higher AhR affinity of BaP in comparison to I3C. Mapping of known aryl hydrocarbon response element (AHRE) sequence motifs in identified DEGs revealed more than 200 genes not possessing any AHRE, and therefore being not eligible for canonical regulation. Bioinformatic approaches modeled a central role of type I and type II interferons in the regulation of those genes. Additionally, RT-qPCR and ELISA confirmed a AhR-dependent expressional induction and AhR-dependent secretion of IFN-γ in response to BaP exposure, suggesting an auto- or paracrine activation pathway of Mϕ.

## 1 Introduction

The aryl hydrocarbon receptor (AhR) is a ligand-activated basic helix-loop-helix transcription factor belonging to the Per-Arnt-Sim family (1). Upon ligand binding, the AhR translocates into the nucleus, where it dimerizes with the AhR nuclear translocator and binds to an aryl hydrocarbon response element (AHRE; also known as xenobiotic response element, XRE) in the promoter regions of a broad range of target genes, among them are several genes encoding xenobiotic-metabolizing enzymes, such as cytochrome P450 (CYP) 1A1 and CYP 1B1 (2).

Apart from its role in detoxification of xenobiotics, i.e., 2,3,7,8-tetrachlorodibenzo-p-dioxin (TCDD) and benzo[*a*]pyrene (BaP), through the induction of CYP monooxygenases and circadian rhythm, AhR is of crucial importance in liver homeostasis, cell cycle control and immune regulation (3–8). The constitutive expression in innate immune cells, i.e., macrophages (Mϕ) and dendritic cells (DC), or type-3 innate lymphoid cells, implies a central function of AhR in innate immune regulation (9). This was supported by the observation that AhR expression is increased during inflammation in human DC or murine Mϕ (10, 11). Moreover, exogenous xenobiotic (e.g., BaP) or nutritional (e.g., indole-3-carbinole, I3C) but also endogenous AhR ligands (e.g., kynurenine, a product of tryptophan metabolism) can modulate innate and adaptive immunity, potentially resulting in either increased or decreased susceptibility to infection or cancer and may trigger autoimmune disorders and allergies (12). Since agonistic and antagonistic AhR ligands may cause different, sometimes contrary, cell- and tissue-specific effects in diseases, the AhR is considered as a promising drug target.

The immunoregulatory function of AhR seems to be realized through both, the canonical and several recently discovered non-canonical AhR signaling pathways (13). The latter are realized at the genomic level through association with other transcription factors, i.e., signal transducer and activator of transcription 1 (STAT1) or nuclear factor-κB (NF-κB) subunits RelA and RelB, causing activation or repression of several target genes such as *c-myc* or *Il6*, respectively (14–19). But also at the non-genomic level non-canonical AhR signaling pathways may be implemented by regulating protein ubiquitination, e.g., as a ligand-dependent E3 ubiquitin ligase (i.e., CUL4^AhR^ complex), targeting substrate proteins for proteasomal degradation such as steroid receptors (i.e., estrogen receptor α (ERα), ERβ, and androgen receptor) (20) and phosphorylation, such as the release of the Src kinase from the cytosolic AhR complex in an active form (4, 21, 22).

In terms of murine Mϕ, surface expression of MHC-II, CD64, CD14, and CD86 as well as cytokine secretion could be modulated through AhR activation only upon stimulation via pattern recognition receptors (PRRs) by pathogen-associated molecular patterns (PAMPs), such as lipopolysaccharide (LPS) or formulations including several PAMPs, such as heat-killed bacteria (10). Moreover, AhR activation interferes with the differentiation of bone marrow-derived myeloid precursors into mature Mϕ (23). Interestingly, AhR activation seems to induce an anti-inflammatory phenotype in PAMPs-activated murine Mϕ, that is characterized by significant upregulation of interleukin (IL)-10 but downregulation of proinflammatory cytokines, such as TNF-α, IL-1β, IL-6, and IL-12 (11). Additionally, AhR activation is linked to interferon (IFN)-α and IFN-γ expression (24, 25). However, it remains unclear so far whether high-affinity vs. low-affinity AhR ligands may activate the same gene expression pattern in Mϕ and other innate immune cells, and thus may have the same impact on innate immune regulation. In this context, also the importance of the source of AhR ligands (xenobiotic vs. nutritional vs. endogenous) is still matter of debate. Therefore, we intended to compare the global gene expression pattern in murine PAMPs-activated bone marrow-derived macrophages (BMMs) following exposure to the AhR ligands BaP (high affinity) or I3C (low affinity) by means of mRNA sequencing.

We identified more than 1,000 differentially expressed AhR-dependent genes that could be related to various functions ranging from basal biological processes to the modulation of innate immunity. Based on computational modeling approaches, the majority of those genes is anticipated to be under canonical control. However, 203 genes do not possess any known AHRE motif, and thus are supposed to be non-canonically or secondarily regulated by AhR. The most interesting cluster of non-canonical signaling concerns to type I and type II interferons. Computational modeling and experimental verification suggest an AhR-mediated induction of IFN-α/β and IFN-γ secretion resulting in autocrine/paracrine Mϕ activation. Of note, those IFN-triggered effects were only observed in BaP- but not I3C-exposed BMMs, suggesting that they were induced ligand-specifically or, more likely, in dependence on the ligand’s affinity and effective concentration.

## 2 Materials and methods

### 2.1 Chemicals and reagents

All chemicals or reagents were obtained from Sigma Aldrich (Taufkirchen, Germany) unless noted otherwise. Cell culture flasks and plates were purchased from Greiner Bio-One (Frickenhausen, Germany). Roswell Park Memorial Institute (RPMI) 1640 culture medium was supplemented with 10 mM 2-[4-(2-hydroxyethyl)piperazin-1-yl]ethanesulfonic acid (HEPES) buffer, 2 mM L-glutamine, 10% (v/v) fetal bovine serum (FBS) and 100 U/ml penicillin, 100 µg/ml streptomycin (Biochrom, Berlin, Germany or PAN-Biotech, Aidenbach, Germany), and 50 μM β-mercaptoethanol to be used as complete cell culture medium. BaP and I3C were dissolved in dimethyl sulfoxide (DMSO).

### 2.2 Mice

Female wild-type (*Ahr^+/+^*) C57BL/6JRj mice were originally purchased from Janvier Labs (St. Berthevin Cedex, France). C57BL/6 AhR knockout (*Ahr* ^-/-^) mice (26) were originally purchased from Jackson Laboratory (Bar Harbor, USA) and back crossed in-house to *Ahr^+/+^* C57BL/6JRj mice for several times, while the *Ahr* deletion was confirmed by genotyping by means of PCR. Animals were used at 8-12 weeks of age for the experiments. Mice were housed as five or six animals per cage in the animal care facility of the Fraunhofer Institute for Cell Therapy and Immunology (Leipzig, Germany) in a temperature- and humidity-controlled room (23 °C, 50 % humidity) under specific pathogen-free conditions with 12 h/12 h of light/dark cycle and free access to pelleted standard rodent chow and water *ad libitum*. All experiments involving laboratory animals had been conducted according to the European Communities Council Directive (86/609/EEC) and were approved by local authorities (registration no. T 10/17, Landesdirektion Sachsen, Leipzig, Germany). All animals were sacrificed using flow-controlled carbon dioxide (1 L/min). All efforts were made to minimize suffering of the animals.

### 2.3 Generation and stimulation of murine bone marrow-derived macrophages

Bone marrow cells were isolated from femur and tibia of each four *Ahr^+/+^* and *Ahr* ^-/-^ mice by flushing with phosphate-buffered saline and harvested by centrifugation (260 x g, 10 min, room temperature, RT). For differentiation, the cells were plated at a density of 4 x 10^5^/ml into tissue culture dishes in RPMI 1640 medium without phenol red (20% FBS, 2 mM L-glutamine, 10 mM HEPES, 100 U/ml penicillin, 100 µg/ml streptomycin, 50 μM β-mercaptoethanol) supplemented with 30 % macrophage colony-stimulating factor-enriched medium (culture supernatant from the fibroblastic cell line L929) prepared in-house as previously described (11). Fresh medium containing the same ingredients was added after 3 days. At day 6 of the myeloid differentiation process adherent cells were scraped off and tested for the surface expression of CD11b (Miltenyi Biotec, Bergisch-Gladbach, Germany) and F4/80 (Miltenyi Biotec) by flow cytometry (CytoFLEX, Beckman Coulter, Krefeld, Germany) in order to verify the differentiation to BMMs. Cells were harvested for further experiments if they revealed more than 80 % expression of CD11b and F4/80. For RNA sequencing experiments, 1 x 10^6^ BMMs/ml were cultured in complete RPMI 1640 medium without phenol red in the presence of 1 µM BaP, 10 µM I3C or 0.01 % (v/v) DMSO (vehicle control) for 6 h. Subsequently, the BMMs were activated for 3 h or 20 h by adding 1 x 10^7^ heat-killed *Salmonella enterica* Serovar Enteritidis (hk *S.*E.)/ml to each culture. Hk *S*.E. that represent an excellent source of several PAMPs for broad-spectrum PRR activation were prepared from a *S.*E. vaccine strain that was kindly provided from IDT Biologika (Dessau-Roßlau, Germany) as previously described (27). For quantitative RT-PCR (*Il10, Il1b, Il12rb1, Slpi, Ido2, Gsta3, Spn and Igf1r*), BMMs were exposed to increasing doses of BaP and I3C in the range of 0.5–100 µM and for *Ifng* expression analyses, BMMs were exposed to 8 nM or 800 nM BaP.

### 2.4 Quantification of mRNA expression by real-time RT-PCR

Total RNA was isolated with TriReagent^®^ according to manufacturer’s instructions (Sigma Aldrich). After quantifying by absorbance at 260 nm/280 nm DNA contamination was degraded by DNaseI (Fermentas, St. Leon Rot, France). The single strand cDNA synthesis was performed using the Transcriptor First Strand cDNA Synthesis kit (Roche Diagnostics, Mannheim, Germany). Optimal primer design and the Universal ProbeLibrary^®^ (UPL; Roche Life Science, Penzberg, Germany) probe selection was performed using the web-based software tool ProbeFinder (Roche) (**Table 1**). Designed primers were purchased from TibMolBiol (Berlin, Germany) and the real-time RT-PCR was performed using the LightCycler^®^ 480 instrument (Roche). PCR assays were prepared using LightCycler^®^ Probes Master kit (Roche) and the appropriate UPL probe with optimal primers.

**Table 1:** Forward and reverse primer sequences for real-time PCR.

Gene	Forward primer 5’→3’	Reverse primer 5’→3’
*Hprt*	tcctcctcagaccgctttt	cctggttcatcatcgctaatc
*Alas1*	ccctccagccaatgagaa	gtgccatctgggactcgt
*Il10*	gctcctagagctgcggact	tgttgtccagctggtccttt
*Il1b*	ttgacggaccccaaaagat	gaagctggatgctctcatctg
*Slpi*	cttgctctggggatcctg	ggctccgattttgatagcat
*Gsta3*	caacttccctctcctgaaagc	caacacattttgcgtcatca
*Igf1r*	agtccctcaaggatggtgtct	cgatctcccagaggacgac
*Il12rb1*	cagggaccagcaaacacat	accagggtctccctagaagc
*Ido2*	gctatcaccatgggattcgt	aagagatcttggcagcacct
*Spn*	gccctgtgccttaaccatt	gaaggtgcaaggccatctc

### 2.5 RNA sequencing library construction

Total RNA was isolated with TriReagent^®^ according to manufacturer’s instructions (Sigma Aldrich). To eliminate all traces of genomic DNA DNAse-digestion (TURBO DNA free Kit, Invitrogen, Thermo Fisher Scientific, Darmstadt, Germany) was performed twice in each sample. Extracted RNA was quantified using a Qubit RNA-Kit and the DeNovix instrument (Biozym, Oldendorf, Germany). Quality of RNA was analyzed by means of a Bioanalyzer 2100 instrument (Agilent Technologies, Waldbronn, Germany).

For subsequent RNA sequencing analyses 500 ng total RNA per sample was used. Library preparation was conducted using TruSeq-Stranded mRNA Sample Prep kit (Illumina, San Diego, CA, USA) according to the manufacturer’s protocol. Fragmentation step was performed for 8 min, following Illumina’s recommendation for high quality input RNA. Quality and quantity of each prepared library was analyzed with the DeNovix instrument (Qubit DNA-Kit) and the Bioanalyzer 2100 instrument (Agilent Technologies). Molarity of each library was calculated and equal amounts were pooled and used for subsequent sequencing (12 pM). Sequencing was performed with 2 x 126-bp paired-end reads using SBS V4 chemistry on a HiSeq 2500 instrument (Illumina). One flow-cell containing 8 lanes was sequenced with 64 pooled libraries.

### 2.6 Computational analysis of RNA sequencing data

Reads were demultiplexed by Illumina’s bcl2fastq (v2.19.0.316). Adapter sequences were removed from reads using adapter removal (v2.2.1a) using parameters –trimns, –trimqualities, –minquality ‘20’, and –minlength ‘30’). HISAT2 (v2.1.0) with parameters –fr and rna-strandness: RF was used to align reads against the mouse genome mm10 (GENCODE release M22) (28, 29). Number of reads per gene were counted by htseq-count (v0.9.1) using parameters –mode intersection-strict, –stranded reverse and –type exon (30). These steps were orchestrated by the workflow-manager uap (31).

Gene set enrichment analysis (GSEA) of whole expression profiles was performed using the GSEA software (v4.1.0, Broad Institute, Cambridge, MA, USA) and the implemented tool “GSEAPreranked” (32). Genes were ranked by log_2_-fold change and false discovery rate (FDR). Gene symbols were not collapsed and default parameters were applied. MSigDB gene set databases “c2.cp.reactome”, “c5.go.bp” were used and complemented with “WP_ARYL_HYDROCARBON_RECEPTOR_ PATHWAY_WP2873” from “c2.cp.wikipathways” (all v7.5.1) (33). Gene sets with FDR < 0.25 and normalized enrichment score (NES) > 1.5 in at least one ligand/time combination were considered for further analysis.

Genome-wide screening for known aryl hydrocarbon response elements (AHRE) in gene promoter regions (3,000 nt upstream of transcriptional start site on both strands) was performed using genome-scale dna-pattern matching function of the Regulatory Sequence Analysis Tools program (RSAT) (34). Known AHRE sequences (AHRE I: 5’-GCGTG-3’, AHRE II: 5’ CATGnnnnnnC(A/T)TG-3’ and RelB AHRE: 5’-GGGTGCAT-3’ were searched against the full length C57BL/6 mouse genome (GRCm38, mm10). Only perfect matches were considered and hits were mapped to next generation sequencing results (**Supplementary Table 1**).

For upstream regulator analysis, RNA sequencing profiles were imported to Ingenuity Pathway Analysis (IPA) software (35) and filtered for AhR-dependent differentially expressed genes (DEGs). Gained upstream regulators were filtered by activation or inhibition (absolute z-score > 2) and targeting AhR-dependent DEGs not possessing any AHRE motif. Functional network analysis was performed using the IPA software. Therefore, all filtered upstream regulators were connected interferon-γ (IFN-γ)-centrically based on IPA knowledge base. Elements, that were computed upstream of direct IFN-γ interactions were subsequently connected by Path Explorer.

### 2.7 Analysis of interferon-γ secretion by ELISA

IFN-γ secretion of BMM was assessed by determination of its concentration in cell culture supernatants by enzyme-linked immunosorbent assay (Mouse IFNg ‘Femto-HS’ High Sensitivity Uncoated ELISA, Invitrogen, Thermo Fisher Scientific) following the manufacture’s protocol. Briefly, 96-well plates were coated with capture antibody overnight at 4 °C. Subsequently after blocking, 100 µl of cell culture supernatants, standards and blanks were added and incubated overnight at 4 °C. Next, samples were incubated with detection antibody for 1 h at RT, prior addition of streptavidin-coupled horseradish peroxidase and incubation for 30 min at RT. Ultimately, detection was started by adding tetramethylbenzidine substrate solution for 15 min at RT and stopped by 1 M phosphoric acid. Washing of 96-well plate was included between all described steps. Finally, the optical density (OD) at 450 nm was measured and cytokine concentration was calculated from a calibration curve achieved with a recombinant mouse IFN-γ standard.

### 2.8 Statistical analysis

Differential gene expression analysis was performed using DESeq2 (v1.28.1) (36). Raw read counts were normalized and variance stabilized. FDR was controlled by Benjamini-Hochberg adjustment. DEGs were filtered by FDR < 0.01. Genes that were filtered as DEGs specifically expressed in either *Ahr^+/+^* or *Ahr* ^-/-^ Mϕ under exposure with AhR ligand and hk *S*.E. were assigned as AhR-dependently affected, whereas genes filtered as DEGs expressed in both *Ahr^+/+^* and *Ahr* ^-/-^ cells were assigned as AhR-independently affected. Genes filtered as DEGs in *Ahr^+/+^* or *Ahr* ^-/-^ Mϕ without AhR ligand exposure where considered as unspecific effects, and therefore excluded. Correlation analyses of gene expression profiles were performed using *cor.test* function in R base environment.

For statistical analyses of RT-PCR experiments, all targeted genes were normalized to housekeeping genes aminolevulinate synthase 1 (*Alas1*) and hypoxanthine-guanine phosphoribosyl transferase (*Hprt*). Relative quantification was performed by means of the LightCycler^®^ 480 software v.1.5 (Roche). Significance of altered relative expression against DMSO-treated sample was determined by Student’s *t*-test (two-sided, unpaired).

For statistical analyses of ELISA experiments, determined ODs were reduced by mean blank values. Calibration curve was calculated from mean values of log_2_-titrated IFN-γ standard applying four-parameter Marquardt regression. Concentrations of IFN-γ in cell culture supernatants were calculated from fitting to calibration curve. Significance of altered IFN-γ secretion against vehicle control (DMSO) was determined by Student’s *t*-test (two-sided, unpaired).

## 3 Results

### 3.1 AhR activation modulates a broad range of gene expression in BMMs

Mϕ are one key player in the innate immune response. To elucidate molecular effects of AhR stimulation on their activation, murine BMMs were exposed to BaP *in vitro*. Subsequently, conditioned BMMs were activated by hk *S*.E. for 3 h or 20 h. To determine the therapeutic potential of targeting AhR and to identify ligand-dependency of downstream effects, BMMs were also exposed to the non-toxic AhR-ligand I3C analogously in the same experimental setup.

**Figure 1:**
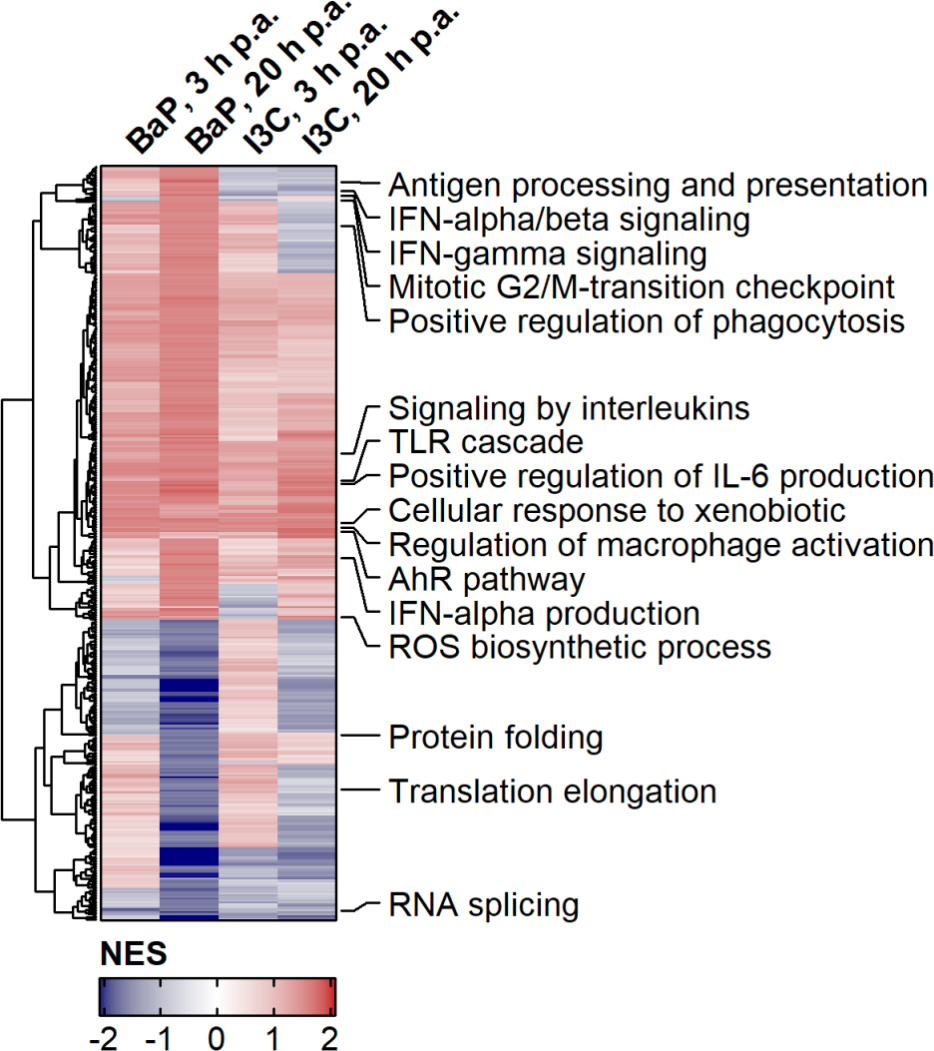
Effects of AhR activation on gene expression profiles during PAMP-induced BMM activation. Murine *Ahr^+/+^* BMMs were exposed to AhR ligands (BaP or I3C) or treated with vehicle control (DMSO) for 6 h (n = 4). Subsequently, cells were activated with hk *S*.E. for 3 h or 20 h (p.a. – post administration) for PAMP activation. Whole cellular RNA was extracted and analyzed by means of RNA sequencing. Relative quantitative whole gene expression profiles of ligand-exposed vs. DMSO-treated cells retrieved from DESeq2 analysis were inspected via gene set enrichment analysis against biological process category of gene ontology and canonical pathways of databases REACTOME and Wiki Pathways. NES of gene sets were used for Euclidean Clustering. Gene sets were selected to cover innate immunity and representative basal cellular processes and annotated in die figure. Complete results are provided as **Supplementary Table 2**.

Overall the expression of 14,549 quality filtered genes was profiled (**Supplementary Table 1**). To elucidate the modulation of gene expression by AhR ligands, whole expression patterns of pairwise-comparisons of ligand-exposed and hk *S*.E.-activated to non-exposed and hk *S*.E.-activated *Ahr^+/+^* BMMs were analysed by gene set enrichment analysis (GSEA). Overall, 467 gene sets were identified as significantly enriched in any ligand/time point combination (**Figure 1**; **Supplementary Table 2**). 179 gene sets were constitutively upregulated by means of positive NES. This includes genes of the “AhR pathway” and in “Cellular response to xenobiotics” as proofs of successful AhR activation upon ligand binding. Additionally, genes of the “Regulation of Mϕ activation”, “Toll-like receptor (TLR) cascade”, “Signaling by interleukins”, “Positive regulation of IL-6 production” and “IFN-α production” were increased in expression by AhR activation. In contrast, 19 gene sets were constitutively downregulated by AhR ligand exposure mainly comprising genes in the transcription and translation process, e.g., “RNA splicing”. Further gene sets were identified with a time-specific expression profile. Whilst genes of “Mitotic G2/M-transition checkpoint” were upregulated, genes involved in “Translation elongation” were downregulated at 20 h compared to 3 h post hk *S*.E. administration. Even more, gene sets with ligand-specific expression profiles were identified. Genes involved in “Protein folding” were upregulated by I3C compared to BaP, but genes of “Antigen processing and presentation” as well as genes involved in both “Signaling by IFN-α/β” and “Signaling by IFN-γ” were upregulated by BaP compared to I3C. Other gene sets, e.g., of “ROS biosynthetic process” and “Positive regulation of phagocytosis” possess ligand and time dependency of expression profiles. In summary, AhR activation modulates the expression of a broad spectrum of genes that are involved in basal cellular processes and innate immunity in a partly ligand-specific manner.

### 3.2 Majority of affected gene expression is AhR-dependent

**Figure 2:**
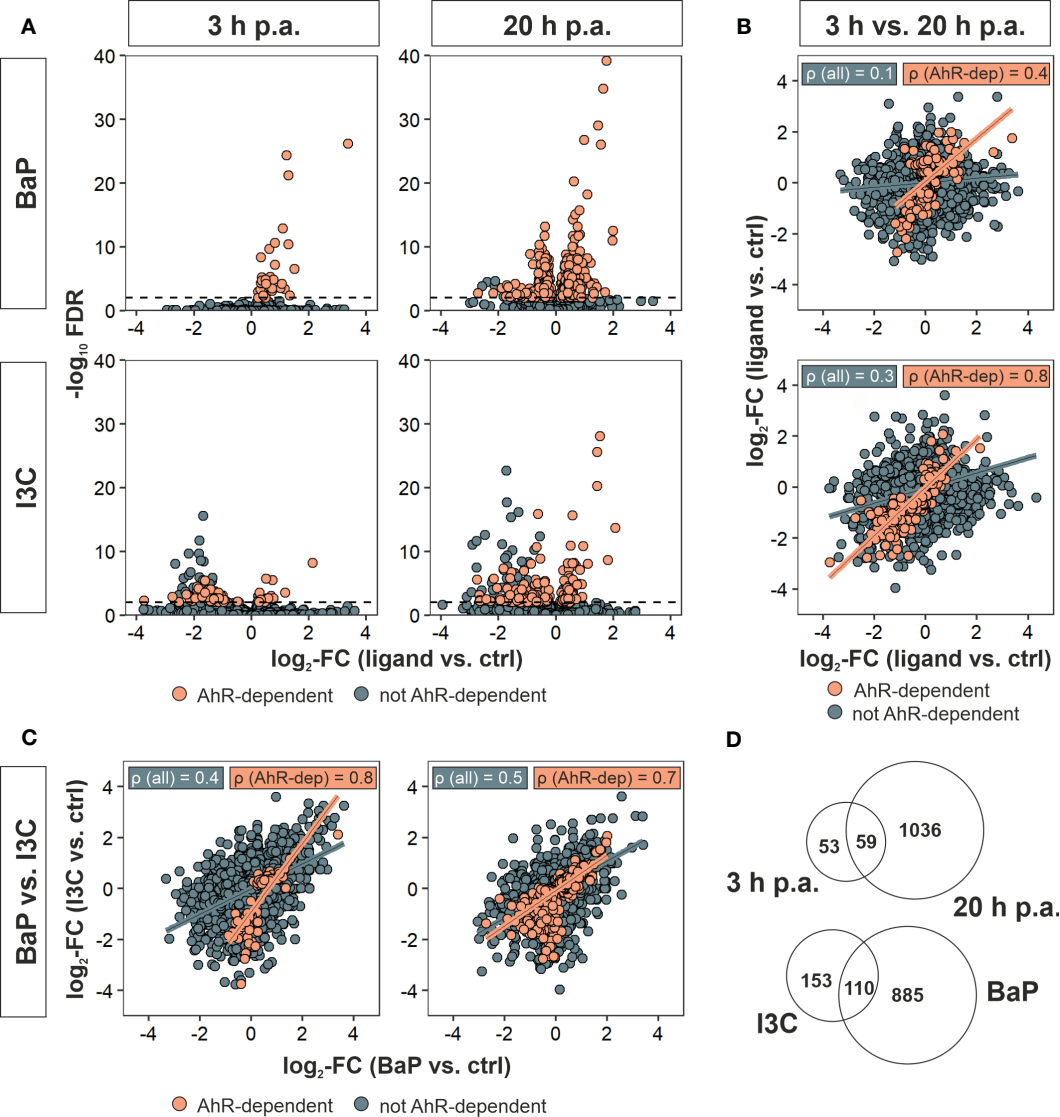
Identification of AhR-dependently differentially expressed genes. Murine *Ahr^+/+^* BMMs were exposed to AhR ligands (BaP or I3C) or treated with vehicle control (DMSO) for 6 h (n = 4). Subsequently, cells were activated with hk *S*.E. for 3 h or 20 h (p.a. – post administration) for PAMP activation. Whole cellular RNA was extracted and analyzed by means of RNA sequencing. Mapped read counts were analyzed by DESeq2 to identify DEGs (n = 4). **(A)** Volcano plots representing log_2_-transformed fold changes (FCs) of gene expression after ligand exposure compared to DMSO-treated BMMs and −log_10_-transformed adjusted *p* values (FDR). **(B)** Correlation of gene expression 3 h to 20 h post administration (p.a.) of hk* S.*E. for BaP (upper panel) and I3C exposure (lower panel) represented by log_2_-FCs. **(C)** Correlation of gene expression after BaP (x-axis) to I3C (y-axis) exposure after 3 h (left panel) and 20 h p.a. of hk* S.*E. (right panel) represented by log_2_-FCs. Linear regression is represented by correlation of all genes (blue) and AhR-dependently DEGs (AhR-dep, orange). Corresponding Pearson’s correlation coefficients (ρ) are indicated in the plots. Complete statistics of correlation analyses are provided as **Supplementary Table 3**. **(D)** Venn-Euler diagrams of total AhR-dependent DEGs comparing 3 h and 20 h p.a. of hk* S.*E. (upper panel) or I3C and BaP exposure (lower panel).

In order to focus on single gene expression and to exclude effects that are triggered by ligand exposure but are independent of AhR, the above-mentioned experimental setup was likewise applied for *Ahr^+/+^* as well as *Ahr^-/-^* BMMs and DEGs of ligand exposed to vehicle control treated BMMs were identified. Only genes that were identified as DEGs in *Ahr^+/+^* or *Ahr^-/-^* Mϕ but not in both were referred to as AhR-dependent DEGs. Out of 1,197 DEGs in total were found after AhR ligand exposure, 1,108 DEGs could be identified to be AhR-dependently affected. However, the number of AhR-dependent DEGs varied between the both ligands and duration of hk *S*.E. activation with more genes being affected by BaP and after 20 h of Mϕ activation via PRRs by PAMPs (i.e., hk *S.*E.), respectively (**Figure 2A**; **Table 2**).

**Table 2:** Number of AhR-dependently differentially expressed genes (DEGs).

Ligand	Duration of BMM activation through hk *S*.E	
	3 h	20 h
BaP	54	972
I3C	73	221

DEGs were filtered by FDR < 0.01. AhR dependencies were elucidated by comparative analysis of *Ahr^+/+^* and *Ahr^-/-^* BMMs.

Only 54 and 73 genes were AhR-dependently modulated after 3 h of BMM activation through hk *S*.E. While all of them are upregulated after BaP exposure, both down- and upregulated genes are present after I3C exposure. After 20 h of hk *S*.E.-induced Mϕ activation, the number of AhR-dependently modulated genes increased up to 972 or 221 upon BaP or I3C exposure, respectively. Pearson’s correlation of gene expression profiles at 3 h to 20 h post hk *S*.E. administration revealed correlation coefficients (ρ) of 0.1 and 0.3 for BaP and I3C, respectively (**Figure 2B**). However, when including only AhR-dependent genes ρ increases to 0.4 and 0.8, respectively. Correlations of transcriptome profiles of BaP- to I3C-exposed BMMs resulted in ρ = 0.4 or 0.5 at 3 h or 20 h post hk *S*.E. administration, respectively (**Figure 2C**). Similarly, when including only AhR-dependent genes, ρ increased to 0.8 and 0.7, respectively. Thus, similar AhR-dependent effects regardless the ligand used for AhR activation may be anticipated. Interestingly, the increment of the resulting regression function is 0.5 and 0.6, which may indicate an affinity-dependent effect. All tested correlations possess significance with p < 2.2 × 10^-16^ (**Supplementary Table 3**). However, in direct comparisons of identified DEGs at specific ligand exposures and time post hk *S*.E. administrations, only 110 AhR-dependent DEGs are common to BaP and I3C exposure, while 885 and 153 genes are affected specifically by one of the both ligands, respectively. Fifty-nine AhR-dependent DEGs were altered at both time points, whilst 53 and 1,036 genes were affected only at 3 h or 20 h post hk *S*.E. administration, respectively (**Figure 2D**).

Hence, whole expression profiles and correlation analyses may point to general AhR-dependent effects that occur at different effect strengths upon the usage of BaP or I3C. However, focussing on single gene expression may allow the identification of ligand-specific target genes.

### 3.3 AhR activation regulates gene expression related to innate immunity

**Figure 3:**
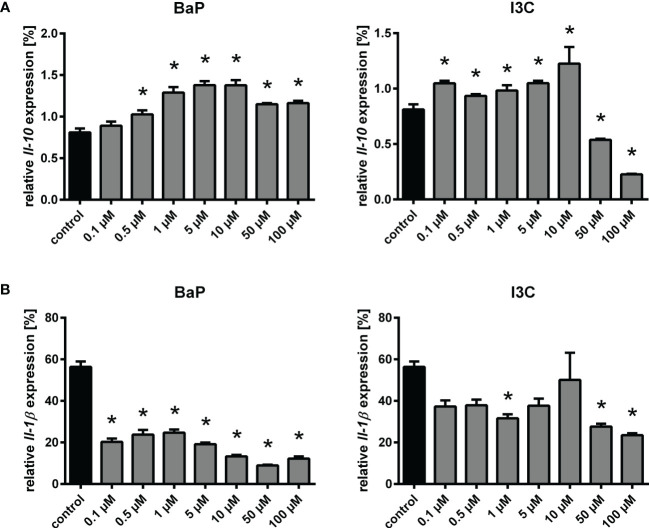
Changes in relative *Il10* **(A)** and *Il1b* **(B)** expression following exposure to different concentrations of BaP or I3C. BMMs from *Ahr^+/+^* mice were treated with indicated concentrations of BaP or I3C or vehicle control (DMSO) for 6 h. Subsequently, cells were activated with hk *S*.E. for 20 h (p.a. – post administration) for PAMP activation. Changes in gene transcription were assessed by real-time RT-PCR. Data represent the mean of the relative cytokine gene expression ± SEM after normalization with housekeeping genes *Alas1* and *Hprt* (n=4). *p ≤ 0.05 indicates significant differences between treated and untreated cells.

Among the AhR-dependent DEG, previously described and putative novel AhR-regulated genes were identified, including canonical and non-canonical targets (**Table 3**). The expression of the canonical AhR-targets *Ahrr* and *Nqo1* was increased by both ligands at both time points in *Ahr^+/+^* but not *Ahr^-/-^* BMMs, confirming successful activation of AhR in *Ahr^+/+^* and no AhR signaling in Mϕ without functional AhR. Interestingly, the transcriptional activation of Glutathione S transferase alpha 3 (*Gsta3*) was slightly induced only by I3C. Expression of *Cyp* genes was not induced, but possesses overall low expression rates near the detection limit. Matrix metalloproteinases (MMP) gene expression was found to be upregulated for *Mmp8* and *Mmp27* by both ligands BaP and I3C. Similar evidence was gained for Kunitz type 1 serine protease inhibitor (*Spint1*), whereas secretory leukocyte protease inhibitor (*Slpi*) expression was only induced by I3C. Kynurenine-producing indoleamine 2,3-dioxygenase 2 (*Ido2*) expression was induced only by BaP. However, the expression of the IDO-regulator interferon regulatory factor 1 (*Irf1*) was induced by both ligands. The transcriptional rates of antibacterial interferon-inducible GTPase 1 (*Iigp1*) and interferon-induced GTP-binding protein (*Mx1*) were found to be increased only by BaP. Similar evidence of a BaP-specific expressional induction was gained for cyclic GMP-AMP synthase (*Cgas*) as well as for the type I interferon receptor alpha and beta chains (*Ifnar1*, *Ifnar2*) Moreover, several important functional Mϕ genes were found to be induced following BaP exposure. Among them are the genes encoding for CD64 (*Fcgr1*), CD16 (*Fcgr3*), CD84 (*Cd84*), toll-like receptor 9 (*Tlr9*), and IL-12Rβ1 subunit (*Il12rb1*). The transcription of the IL-21R (*Il21r*) was upregulated by both ligands, BaP and I3C. Interestingly, *Spn* a gene encoding for sialophorin (leukosialin, CD43) a major sialoglycoprotein expressed on Mϕ but also several other leukocytes, mainly T lymphocytes, was found to be downregulated following BaP exposure. Unexpectedly, no significant changes in *Il1b* and *Il10* transcriptional rates could be detected, although trends were observable for reduction of *Il1b* but induction of *Il10* gene expression through BaP exposure (**Supplementary Table 1**). However, verification of those results by RT-qPCR revealed a significant and dose-dependent reduction of *Il1b* but induction of *Il10* mRNA expression through exposure with both ligands, BaP and I3C (**Figure 3**). In terms of chemokines, transcription was reduced in case of CC-chemokine ligand *Ccl3* but induced in case of *Ccl7* following BaP exposure. Finally, the expression of insulin-like growth factor 1 receptor (*Igf1r*) was declined by I3C, whilst its ligand *Igf1* was induced by BaP.

**Table 3:** Selected AhR-dependently differentially expressed genes.

Gene	BaP				I3C				AHRE
	3 h p.a.		20 h p.a.		3 h p.a.		20 h p.a.		
	*Ahr^+/+^*	*Ahr^-/-^*	*Ahr^+/+^*	*Ahr^-/-^*	*Ahr^+/+^*	*Ahr^-/-^*	*Ahr^+/+^*	*Ahr^-/-^*	
*Ahrr*	**3.37**	-0.35	**1.76**	0.31	**2.13**	0.35	**1.53**	-0.13	Yes
*Nqo1*	**0.72**	0.17	**0.74**	0.08	**0.69**	0.33	**1.12**	0.08	Yes
*Gsta3*	-0.19	0.16	0.16	0.19	0.16	0.18	**0.59**	0.51	Yes
*Mmp8*	**0.82**	0.01	**1.65**	-0.30	**0.54**	0.12	**1.45**	-0.55	Yes
*Mmp27*	0.50	0.17	**1.97**	-0.76	0.22	-0.14	**1.81**	-0.19	**No**
*Spint1*	**1.50**	1.59	**1.56**	0.34	**1.18**	0.94	**1.43**	-0.31	Yes
*Slpi*	0.15	0.05	0.07	0.09	0.11	0.11	**0.44**	0.37	Yes
*Ido2*	**1.17**	0.12	**1.22**	0.12	0.54	-0.14	0.77	-0.02	Yes
*Irf1*	0.12	0.02	**0.24**	0.05	0.11	0.08	**0.26**	0.10	Yes
*Iigp1*	-0.09	-0.46	**0.79**	-0.24	0.01	-0.16	0.46	-0.23	**No**
*Mx1*	-0.06	-0.31	**0.59**	-0.18	-0.01	-0.17	0.24	0.02	**No**
*Ifnar1*	-0.01	-0.02	**0.19**	0.03	0.01	-0.01	0.10	-0.02	Yes
*Ifnar2*	0.02	-0.01	**0.17**	0.01	0.03	0.01	0.10	-0.01	Yes
*Cgas*	-0.01	-0.21	**0.29**	-0.02	0.02	-0.13	0.23	0.01	**No**
*Fcgr1*	0.03	-0.08	**0.33**	-0.11	0.02	0.00	0.13	-0.03	**No**
*Fcgr3*	0.08	0.00	**0.45**	0.03	0.08	0.09	0.21	-0.12	Yes
*Cd84*	-0.11	-0.12	**0.37**	0.03	0.00	0.04	0.24	-0.03	**No**
*Spn*	-0.10	0.18	**-0.80**	0.00	-0.03	0.15	-0.33	0.08	Yes
*Tlr9*	0.06	0.14	**0.50**	-0.09	0.18	0.00	0.10	0.13	Yes
*Il12rb1*	0.25	0.47	**0.90**	-0.12	0.21	0.25	0.60	0.28	**No**
*Il21r*	**0.81**	0.03	**0.60**	-0.02	0.40	0.10	**0.48**	0.10	Yes
*Igf1r*	-0.02	0.04	-0.21	-0.02	-0.16	-0.10	**-0.75**	-0.28	Yes
*Igf1*	-0.09	-0.14	**0.34**	0.03	-0.07	-0.09	0.14	0.03	Yes
*Ccl3*	-0.03	0.14	**-0.48**	0.14	-0.03	0.02	-0.33	0.05	Yes
*Ccl7*	-0.07	-0.09	**0.31**	-0.06	-0.22	-0.23	-0.14	-0.22	Yes
*Gprc5b*	0.00	0.15	**0.66**	0.09	0.16	0.01	0.14	0.30	Yes
*Cdk14*	-0.04	-0.25	**0.35**	-0.04	-0.08	-0.23	-0.03	-0.29	Yes

Numbers represent log_2_-tranformed fold changes of expression after ligand stimulation compared to non-exposed state from RNA sequencing experiments. Bold printed numbers indicate significant expression changes (FDR < 0.01). p.a. = time post hk S.E. administration. AHRE, aryl hydrocarbon response element.

Hence, AhR modulates the expression of several genes in part ligand-dependently. Interestingly, however, for most of the genes, that are differentially expressed after BaP but not I3C exposure, the expressional trend could be observed for both ligands, which implies that the observed effects depend on the affinity of the individual AhR ligand. To this end, we selected genes of importance in innate immunity, that were identified as ligand-specifically regulated and performed RT-qPCR experiments to monitor their expressional changes under exposure with different concentrations of both ligands used in this study, i.e., BaP and I3C (**Supplementary Figure 1**). The ligand-specific upregulation of *Gsta3* through I3C observed in the RNA sequencing result was confirmed by the RT-qPCR analysis. Similarly, an expressional repression of *Igf1r* by I3C was confirmed. Interestingly, an expressional activation of *Igf1r* through BaP was revealed by RT-qPCR, that was not anticipated from RNA sequencing experiments. RT-qPCR revealed upregulation of *Il12rb1*, *Ido2*, and *Slpi* expression through both ligands, whereas the RNA sequencing data showed only a differential expression for BaP (*Il12rb1*, *Ido2*) or I3C (*Slpi*). However, expressional trends as observed in RT-qPCR were already anticipated from RNA sequencing indicating a relation of the observations to higher sensitivity and accuracy of RT-qPCR over RNA sequencing. However, in contrast to the RNA sequencing data *Spn* was not downregulated but upregulated in RT-qPCR analysis.

### 3.4 Absence of aryl hydrocarbon response elements reveals non-canonical AhR targets

To model the mechanism of AhR regulation the promotor regions of all AhR-dependent DEG were screened for the known AHRE: AHRE I (5’-GCGTG-3’), AHRE II (5’- CATGnnnnnnC(A/T)TG-3’) and RelB AHRE (5’-GGGTGCAT-3’). Of all 1,108 AhR-dependent DEG, 905 genes (82 %) possess at least one of the AHRE in their promotor regions and can be considered as potential canonical AhR targets. However, the proportions and usage of AHRE motifs vary between the used ligands and duration of hk *S*.E. activation (**Table 4**). Upon BaP exposure, proportion of AhR-affected genes possessing an AHRE I motif is higher compared to I3C exposure at both time points (85 % and 81 % vs. 49 % and 68 %). While under BaP exposure the proportion of AHRE I-possessing genes was higher after 3 h compared to 20 h of BMM activation by hk *S*.E., we found a reciprocal result upon I3C exposure. Similarly, also a higher proportion of AHRE II-possessing genes was observed in response to BaP compared to I3C exposure but without changes between duration of Mϕ activation (18 % vs. 14 %). The proportion of RelB AHRE possessing AhR-dependent DEGs did not vary between both ligands but was higher after 20 h compared to 3 h of Mϕ activation (6 % vs. 3 %). However, although the presence of an AHRE does not automatically point to a canonical regulation of the target gene, the absence of an AHRE excludes it definitely. Thus, the highly regulated genes *Mmp27*, *Iigp1*, *Mx1*, *Fcgr1*, *Cd84*, and *Il12rb1* can be considered as non-canonical targets (**Table 3**).

**Table 4:** Identification of AHRE motifs in AhR-dependently differentially expressed genes.

Ligand	Time p.a.	AHRE I	AHRE II	RelB AHRE
BaP	3 h	28/33 (85 %)	6/33 (18 %)	1/33 (3 %)
	20 h	785/965 (81 %)	174/965 (18 %)	58/965 (6 %)
I3C	3 h	31/63 (49 %)	9/63 (14 %)	2/63 (3 %)
	20 h	137/202 (68 %)	29/202 (14 %)	13/202 (6 %)

Genes filtered as DEG in *Ahr^+/+^* BMMs for both ligands and time post hk S.E. administration (p.a.) were screened for AHRE I, AHRE II, and RelB AHRE binding motifs in their promotor regions. Numbers indicate proportions of motif-possessing genes to all AhR-dependent DEGs.

### 3.5 Computational modelling suggest a central role of interferons in non-canonical signaling

**Figure 4:**
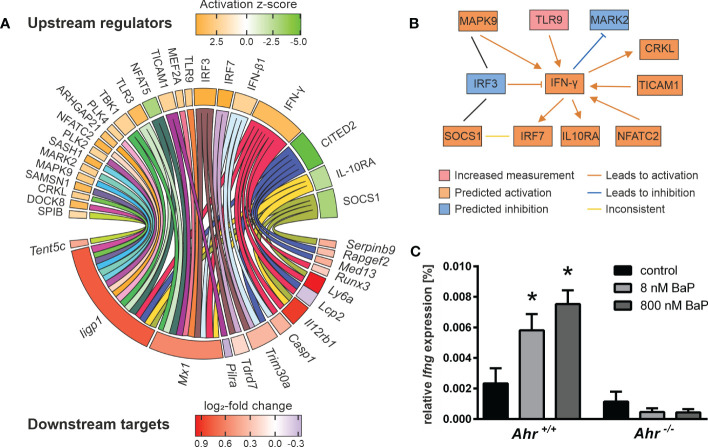
Putative mechanisms in AhR-dependent gene expression. Murine *Ahr^+/+^* BMMs were exposed to BaP or treated with vehicle control (DMSO) for 6 h. Subsequently, cells were activated with hk *S*.E. for 20 h for PAMP activation. Whole cellular RNA was extracted and analyzed by means of RNA sequencing. Mapped read counts were analyzed by DESeq2 to identify DEGs (n = 4). **(A)** Potential upstream regulators were predicted from AhR-dependent gene expression to identify 24 regulator elements (upper panel) that target 14 non-AHRE motif-possessing genes (lower panel). Circle plot illustrating functional interactions of upstream regulators to downstream targets. Outer grid color represents predicted activation z-score (for upstream regulators) and measured log_2_-fold change (for downstream targets). **(B)** Upstream regulator interaction network modified from IPA output (**Supplementary Figure 2**). Nodes represent measured or predicted upstream regulator activities and interactions based on experimental results from BaP-exposed BMMs activated for 20 h with hk *S*.E. **(C)** BMMs from *Ahr^+/+^* and *Ahr^-/-^* mice were exposed to BaP (8 nM or 800 nM) or DMSO (vehicle control) for 6 h. Changes in gene transcription of *Ifng* were assessed by quantitative real-time RT-PCR after activation of BMMs with hk *S*.E. for 20 h. Data represent the mean of the relative expression ± SEM after normalization with housekeeping genes *Alas1* and *Hprt* (n = 3). *p ≤ 0.05 indicates significant differences between treated and untreated cells.

To evaluate mechanisms beyond canonical AhR signaling the expression profiles were filtered for AhR-dependent DEGs and analyzed via IPA to predict the activity of potential upstream regulators. Only profiles of BMMs exposed to BaP and activated for 20 h with hk *S*.E. resulted in sufficient analysis depth and will be focused in the further section. The activity of 36 potential upstream regulators could be modeled (**Supplementary Table 4**). Of those, 24 were found to regulate 14 downstream targets not possessing any of the screened AHRE motifs, and thus cannot be targeted directly by canonical AhR signaling (**Figure 4A**). Among the putative upstream regulators are type I (IFN-β) and type II (IFN-γ) interferons and their associated transcriptional regulators (IRF3, IRF7) as well as transmembrane receptors (IL-10Rα, TLR3, TLR9), kinases (e.g., CRKL, MARK2, MAPK9) and others (e.g., DOCK8, SOCS1, TICAM1). Several proinflammatory regulators (e.g., IFN-γ, IFN-β, TLR3, TLR9, TICAM1) were predicted to be activated. Four of the modeled upstream regulators are predicted to be inhibited. Among those are the anti-inflammatory regulators IL-10Rα, SOCS1 and CITED2. The most non-AHRE motif-possessing AhR-dependent DEGs are known to be directly or indirectly regulated by IFN-γ including *Iigp1*, *Mx1*, *Casp1*, *Il12rb1*, *Lcp2*, *Ly6a*, and *Runx3*. All but *Lcp2* were found in upregulated expression states. Ten out of 14 genes are targeted by two or more upstream regulators. For example, *Iigp1* is regulated by 17 and *Mx1* by 10 upstream elements, representing highly complex regulation mechanisms.

The computed upstream regulators itself are interconnected. Remarkably, a functional network that connects IFN-γ to upstream and downstream effects, is modeled to be activated. The IFN-γ expression and secretion is regulated by TICAM1, TLR9 and NFATC2. IFN-γ itself regulates the activity of MARK2, CRKL, SOCS1, and MAPK9. The latter two interact with IRF3. Further, IFN-γ regulates the expression of IRF3, IRF7, and IL-10Rα. These findings suggest a crucial impact of AhR activation, i.e., through BaP, on IFN-γ production and signaling in Mϕ (**Figure 4B**). *Ifng* itself does not possess any of the screened AHREs and its expression was not detected by RNA sequencing. Due to the ongoing controversy of IFN-γ secretion by Mϕ, IFN-γ transcription in response to AhR ligation was additionally proved by RT-qPCR, revealing AhR- and BaP-dependent transcription of *Ifng* (**Figure 4C**). This result implies IFN-γ synthesis in BMMs, that involves a BaP-triggered, non-canonical AhR-dependent mechanism. In turn, autocrine and paracrine activation of BMMs through IFN-γ may partly explain the gene expression profiles found in this study.

## 4 Discussion

This study complements and extends previous approaches elucidating the molecular mechanisms of AhR-mediated effects by means of (i) comparing *Ahr^+/+^* and *Ahr^-/-^* cells, (ii) comparing prototypic high-affinity and low-affinity ligands, and (iii) transcriptomics in combination with bioinformatic genomic analyses (37–39). The aim of the present study was, to identify potential differences in the global gene expression pattern of activated murine BMMs subsequently to exposure with high-affinity vs. low-affinity AhR ligands represented by BaP and I3C, respectively, by means of mRNA sequencing. As an *in-vitro* model well-established in our laboratory we utilized BMMs from *Ahr^-+/+^* and *Ahr^-/-^* C57BL/6 mice (11, 23). The latter do not possess any remaining AhR activity, and are therefore suitable to study AhR-dependent effects *in vivo* and *in vitro* (26). For our *in-vitro* model, i.e., BMMs from *Ahr^-/-^* mice, functional AhR deficiency was confirmed by absent *Ahrr* expression upon incubation with AhR ligands (data not shown). BMMs were alternatively exposed to both AhR ligands, BaP or I3C, for 6 h prior to either 3- or 20-h activation through a *Salmonella* antigen preparation (i.e., hk *S*.E.), that comprises several PAMPs (i.e., LPS, flagellin, CpG, NOD1/2) and is therefore optimally suitable to stimulate different pattern recognition receptors. Using this approach, we intended to get new insights into ligand-specific molecular effects of latent AhR activation prior to an innate inflammatory response. This in turn should contribute to unravel the mechanism(s) of action how AhR activation may control acute and chronic inflammatory conditions in infection, cancer, or immune-mediated diseases as shown by many authors (38, 40–46).

In order to address the impact of the AhR ligand’s nature, that means high-affinity (i.e., BaP) vs. low-affinity (i.e., I3C) compound, on the AhR-dependent gene expression in Mϕ, we have identified and evaluated DEGs that are specifically expressed in response to either BaP or I3C and, in addition, DEGs that were significantly up- or downregulated by both types of AhR ligands. While the majority of AhR-dependently affected genes (885 DEGs) were found to be affected only by the high-affinity AhR ligand BaP, only 153 DEGs were influenced by the low-affinity AhR ligand I3C. Only a minority of 110 DEGs where affected by both AhR ligands, BaP and I3C. According to our expectations, this result confirmed a stronger influence of the high-affinity AhR ligand onto the functional activity of Mϕ. Interestingly, this result also reveals that high affinity of an AhR ligand is not sufficient to affect the entire potential repertoire of DEGs in Mϕ, suggesting that the individual chemical structure, and thus the individual binding mode in the AhR binding pocket seems to be a critical condition for modifying the expression of certain Mϕ genes.

We identified several DEGs, previously related to innate immunity, that possess known AHRE motifs, and thus are putatively under canonical AhR regulation. Among those genes are *Il21r*, *Spint1*, *Slpi*, *Irf1*, and *Ido2*, that were found to be expressionally activated by both ligands, BaP and I3C, in RNA-seq or RT-qPCR experiments. IL-21 inhibited LPS-induced expression of IL-1β, TNF-α, and IL-6 in peritoneal Mϕ, but not of IFN-γ, IL-10, CCL5, or CXCL2 via inhibiting the phosphorylation of ERK and translocation of NF-κB (47). Further, IL-21R-mediated signaling is involved in M2‐like Mϕ polarization by decreasing the expression of CD86, iNOS, and TLR4 and by increasing STAT3 phosphorylation that promotes the secretion of IL-10 by Mϕ (47–49). *Irf1* expression is regulated by IFN-γ and was previously shown to induce the expression of *Ido2* (50). AhR itself is required to induce *Ido2* expression, that encodes for an immunosuppressive enzyme that catabolizes tryptophan into the endogenous AhR ligand kynurenine. In turn, AhR-mediated increased IDO levels, either directly or indirectly via IRF-1, contribute to decreased innate immunogenicity as previously shown for DC and Mϕ (40, 50). To the best of our knowledge, no AhR-mediated function was previously described for the here observed *Slpi* expressional induction. However, SLPI was shown to inhibit IL-1β maturation in human monocytes further contributing to an AhR-mediated decreased proinflammatory phenotype (51). Interestingly, *Gsta3* and *Igf1r* both possess AHRE-motifs in their promotor regions and were previously shown to be either transcriptionally induced or functionally activated by AhR stimulation (37, 41). Here, we present an I3C-specific induction of *Gsta3* and contrary expressional regulation of *Igf1r*, implying either ligand-specific or further AhR-independent regulatory mechanisms to be involved. Additionally, DEGs not possessing any known AHRE were identified, including *Fcgr1* (encoding for the high-affinity IgG receptor FcγR1/CD64), *Cd84*, and *Il12rb1*. CD64-enriched CD11^+^/F4/80^+^ Mϕ have previously been detected in the peritoneal cavity of BaP-exposed mice in a systemic *Salmonella enterica* infection model (45). *Cd84* was linked to inhibition of IL-1β production contributing to declined proinflammatory effects (52). However, Wang et al. reported an I3C-mediated expressional repression for *Cd84* that was only observable in undifferentiated but not phorbol 12-myristate 13-acetate-induced differentiated THP-1 cells. Here, we point to a BaP-specific expressional induction of *Cd84* in BMMs, implying a species-, cell type- or differentiation state-specific regulation. *Il12rb1* was found to be expressionally activated by both ligands, BaP and I3C, in our study. The mature IL-12Rβ1 acts as subunit in both the IL-12 and IL-23 receptor complexes that bind the corresponding proinflammatory cytokines. IL12Rβ1 deficiency has been associated with childhood-onset and recurrence of salmonellosis, tuberculosis, and candidiasis indicating its role in the prevention of bacterial and fungal infection (53–56). However, to the best of our knowledge, no previous AhR-mediated regulation of *Il12rb1* has been described. It can be speculated, that IL12Rβ1 expression might represent a mechanism for autocrine or paracrine activation of Mϕ resulting in the activation of STAT4 via TYK2 and JAK2 and subsequently in the induction of IFN-γ production; this process might be supported by IL-18 (57).

Overall, we gained evidence, that AhR activation by both ligands, BaP and I3C, reduces proinflammatory effects in PAMP-activated Mϕ. This is in line with previous observations *in vitro* and *in vivo*. BaP seems to dampen acute proinflammatory responses by suppressing proinflammatory cytokines (e.g., IL-1β) but induce anti-inflammatory cytokines (e.g., IL-10), and thus prevent septic shock (11, 45). However, other studies reported increased levels of IL-1β production upon AhR ligand exposure, for example in the human synovial fibroblast cell line MH7A by 3-methylcholanthrene (58) or in the human monocyte cell line THP-1 in response to AhR activation by polychlorinated biphenyls (59). In fact, most studies focus on direct effects of AhR ligands on resting cells. Here, we investigated the modulation of gene expression in PAMP-activated Mϕ. Thus, different observations of IL-1β production rates might be related to activation state of the cell but also to the cell type or species differences. Nonetheless, BaP may also directly or indirectly exacerbate Th2-driven inflammatory responses by suppressing proinflammatory cytokines that induce Th1 responses, i.e., IL-12 (42–44). Another important immunoregulatory effect of BaP concerns to reciprocal regulation of IL-22 and IL-17, suggesting an impact of BaP on the homeostasis at epithelial barriers (60). Notably, these immunomodulatory effects of BaP occur preferentially at lower BaP doses. Previous studies found, that BaP represents a high-affinity ligand with a relative AhR ligand binding affinity of 617 nM (indicated as IC_50_ – competition of [^3^H]TCDD; for comparison: IC_50_ of TCDD and FICZ = 1 nM) while I3C possesses a very low relative binding affinity to AhR (IC_50_ = 26 mM) (61, 62). Moreover, Tagliabue et al. previously proofed, that ligand binding affinity and potency to stimulate AhR nuclear translocation/DNA binding were relatively well correlated (EC_50_) (61). Thus, the stronger effect of BaP in terms of the high number of affected genes found in our study might be due to its higher binding affinity and EC_50_ values compared to I3C, and thus to its higher relative concentration applied in this study. Although, I3C was used at a 10-fold higher concentration than BaP, its affinity to the AhR is estimated to be about 42,000-fold lower compared to BaP resulting in a dose advantage of factor 4,200 as applied for BaP. Given, that the BaP concentration of 1 µM applied in the present study is considered as “low-dose” from previous studies (11, 45), BaP may be seen as a highly potent immunoregulatory AhR ligand. However, genes that are affected either by BaP or I3C might reflect differential responses caused by the individual steric localization of an individual AhR ligand inside the binding cavity of the receptor, as can be presumed from docking studies using different AhR ligands (61) rather than determined by the affinity of the AhR ligand. Hence, further putative AhR ligands, e.g., quercetin (46), that combine non-toxic properties with higher affinities or different binding modes should be tested in future studies for their utilization in immunomodulatory therapies.

In addition to (i) the concentration and (ii) the individual chemical structure of an AhR ligand, the number of AhR-affected genes also critically depends (iii) on the duration of PRR-mediated Mϕ activation. Using a transcriptomics approach, the present study identified 18 times vs. 3 times more DEGs at 20 h vs. 3 h of activation for BaP or I3C, respectively. This implies an even stronger dependency of AhR-driven immunomodulation on the activation state of the Mϕ as already previously shown by our group by investigating the mRNA or protein expression of individual genes of interest using real-time RT-PCR or flow cytometry, respectively (11, 23). This underlines the value of applying untargeted analyses such as transcriptomics in screening approaches. However, the individual nature of the genes differentially regulated at 3 h vs. 20 h of activation revealed different clusters of genes, reflecting specific early or late occurring cellular processes. For example, genes of “Mitotic G2/M-transition checkpoint” were upregulated, whereas genes involved in “Translation elongation” were downregulated at 20 h compared to 3 h of PAMP-induced Mϕ activation. Furthermore, the chemical stability and metabolic turnover of the used ligands are crucial to affect gene expression, especially at different time scales. BaP is chemically inert and requires metabolic activity to be degraded (63). This is mainly achieved by CYP1A1 and NQO1 that were shown to be active in a variety of organoid cultures (64). In the present study, we did not observe an upregulation of *Cyp* expression and overall low expression rates of these main BaP-degrading enzymes. However, *Nqo1* was upregulated in expression by both ligands indicating only a limited degradation of BaP in the here used experimental setup. In contrast, I3C is chemically condensed to e.g., 3,3’-diindolylmethane (DIM) and 5,11-dihydroindolo-[3,2-b]carbazole (ICZ) at acidic conditions, that is mainly achieved in the stomach at *in-vivo* conditions (65). Those metabolites were shown to possess even higher affinity to AhR as I3C itself (62). However, we and others have clearly shown, that I3C induces AhR activation *in vitro* without acidification, likely through the convertion to diindolylmethane (DIM) (66). Although, I3C possesses very low affinity to the AhR it induces several genes via both the canonical and the non-canonical pathway as shown in our work but also by other authors. Thus, it might be anticipated, that the effects observed upon I3C exposure are based on AhR activation by I3C itself as well as its metabolites.

Independent of their nature as AhR ligands, both BaP and I3C affect other pathways and biological processes that are not mediated via the AhR. For instance, BaP was shown to increase TNF-α production in human primary macrophages via ERK1/2 and independent of AhR (67), whereas I3C directly binds to a variety of intracellular receptors, as reviewed previously (68). However, by including *Ahr^-/-^* controls into this study we only reported AhR-dependent effects.

Although multiple AhR-dependent effects were described in the last decades, the diverse modes of action are not yet completely understood. Early attempts applied experimental and *in silico* approaches to screen for canonical targets on whole genome scales. Several hundred putative targets were predicted for human, mouse and rat based on position weight matrices and similarity scores (69). Further, extensive longitudinal expression screening of BaP-exposed cells revealed 81 primary responding genes and more than 1,000 side or secondary effects (70). Those studies found that several transcriptional regulators possess AHRE motifs and primarily respond to AhR activation. Thus, a transcriptional cascade starting at AhR and accounting for multiple phenotypical effects depending on the cell type and activation state was previously proposed. Indeed, AHRE were identified by genome screening in the promotors of the transcription factors CEBP-β, NF-κB1, NF-κB2, NF-κB3, RelB, c-Rel, Jun, IRF-1, IRF-4, and STAT3 (71). Those transcription factors regulate the expression of cytokines in activated Mϕ and may account for the expressional activation of genes involved in the IFN and IL-6 production observed in this study. However, genome-wide screening for known AHRE motifs may not be sufficient as the presence of AhR binding motifs in promotor regions does not necessarily indicates its accessibility for an activated AhR complex, and thus transcriptional activation. Hence, such in-silico screening approaches should be accompanied with advanced bioinformatic approaches for sequence homologies and conservations across different species (72) or by experimental validation applying chromatin immunoprecipitation DNA sequencing (73) in future studies. In contrast to the above-mentioned studies, we did not focus to identify canonical AhR targets based on AHRE motif presence but excluded them to gain insights into non-canonical or secondary effects of AhR in the PRR-mediated Mϕ activation. Therefore, only DEGs not possessing any known AHRE were included for upstream regulator prediction and functional network analysis. Such bioinformatic modeling has previously successfully been applied to identify IRF-7 as a key regulator in LPS-mediated activation in human alveolar Mϕ via TLR4 (74).

Although non-AHRE possessing DEGs account for only a minority of all DEGs (< 20 %), one of the most notable results of the here presented approach concerns to the role of the production and signaling of interferons in the activation of Mϕ in innate immunity. We gained multiple evidence in AhR interference in the release of type I (i.e., IFN-α, IFN-β) and type 2 interferons (i.e., IFN-γ). As such, the genes of the IFN-α/β-inducing *Irf1* and *Cgas* were expressionally increased, whereas IRF3 and IRF7 were modeled to be activated from gene expression profiles upon BaP exposure. In sum, transcriptomics profiles pointed to an induced type I interferon production upon AhR activation as revealed from gene set enrichment analysis. The induction of IFN-α/β expression and secretion by Mϕ after PRR activation by bacterial PAMPs is a well-known concept (75, 76). Here, we propose further evidence of modulation of the type I interferon production through AhR activation. The production of IFN-γ by Mϕ has been a matter of debate until Munder and colleagues delivered convincing evidence that Mϕ are capable of producing IFN-γ in response to combined stimulation with IL-12 and IL-18 (77). Later, Schindler et al. demonstrated that IL-12/IL-18 induced IFN-γ production requires STAT4 signaling (78). It was previously shown that AhR activation modulated the IFN-γ production in Mϕ upon viral infection and autoimmune disease both *ex vivo* and *in vivo* (25, 79). Here, we confirmed an AhR- and ligand-dependent induction of *Ifng* expression by RT-qPCR. The finding that the *Ifng* gene does not possess any of the screened AHREs let assume that AhR ligands may trigger IFN-γ synthesis and secretion in Mϕ via a non-canonical AhR-dependent mechanism. Additionally, we provide evidence for induced autocrine/paracrine effects of the increased production of interferons. For instance, *Ifnar1* and *Ifnar2* were expressionally activated upon AhR activation. Subsequently, IFN-α/β but also IFN-γ signaling was modeled as upregulated based on gene set enrichment and upstream regulator analysis. This was, among others, based on expressional activation of *Iigp*, *Mx1*, *Trim30a*, *Casp1*, *Il12rb1*, *Lcp2*, *Ly6a*, and *Runx3*. None of these genes does possess any of the screened AHRE motifs implying a non-canonical or secondary, autocrine/paracrine interferon-driven effect. An AhR-dependent modulation of IFN-α signaling in antiviral immunity in Mϕ has previously been demonstrated and linked to IRF7 targeting by AhR interacting protein (AIP) (24, 80).

**Figure 5:**
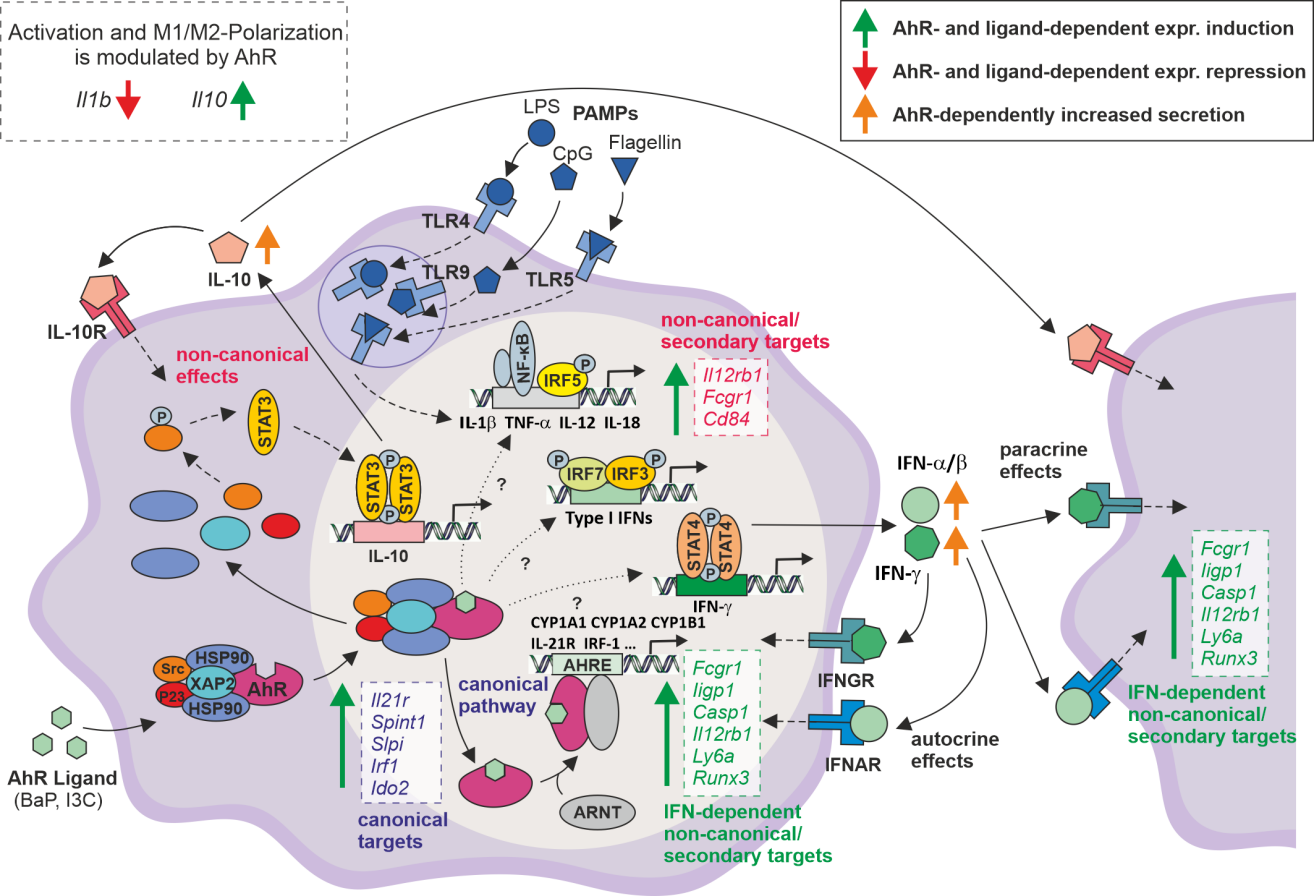
Proposed model for the modulation of PAMP-induced Mϕ activation through AhR ligand exposure. After entering the cytoplasm, AhR ligands such as BaP or I3C bind to a complex of several proteins, containing AhR, HSP90, XAP2, P23, and Src. AhR ligand binding induces a conformational change in this protein complex resulting in translocation of the whole complex into the nucleus where it is degraded to release the AhR molecule. Inside the nucleus, AhR associates with the Aryl hydrocarbon nuclear translocator (ARNT) and binds to the Aryl hydrocarbon response element (AHRE) in the promotor region of target genes, i.e., *Cyp1a1*, *Cyp1a2*, *Cyp1b1*, inducing their transcriptional activity (➪ canonical, genomic targets). This study confirms or suggest the expressional activation of several key immune genes, i.e., *Il21r*, *Irf1*, but also *Spint1*, *Slpi*, and *Ido2* as putative AHRE motif-possessing canonical targets. In the cytoplasm, the AhR complex included src tyrosine kinase phosphorylates STAT3 leading to its dimerization and nuclear translocation. In the nucleus STAT3 binds to a response element in the *Il10* promotor, and thus inducing IL-10 production (81). This is the most prominent non-canonical AhR-dependent pathway. If IL-10 is secreted, it exerts autocrine and paracrine anti-inflammatory effects, leading to the suppression of gene expression of proinflammatory cytokines, i.e., *Il1b*, *Tnfa*, *Il12a*/*b*. Further, this study suggests the AhR-dependent expressional induction of key immune genes for Mϕ activation, such as *Il12rb1*, *Fcgr1*, *Cd84*, that do possess an AHRE motif and thus, are regulated by non-canonical or secondary mechanisms. Of note, from several expressional profiles it is anticipated that the transcriptional activation of type I (IFN-α, IFN-β) and type II (IFN-γ) interferons is regulated by AhR via so far unknown molecular mechanisms possibly including both canonical and non-canonical pathways. Gene expressional profiles and bioinformatic approaches suggest an autocrine/paracrine effect of secreted interferons partly explaining the here presented results. Solid lines ⟹ known mechanisms covered in the figure; dashed lines ⟹ known mechanisms not covered in the figure; dotted line ⟹ unkown mechanisms. Boxes indicate genes expressionally induced in the study.

In conclusion, the here presented study extends the knowledge of molecular effects and mechanisms of AhR activation in the modulation of PAMP-induced Mϕ activation (**Figure 5**). More than 1,000 genes were identified to be modulated in an AhR- and ligand-dependent manner. The majority of those genes, such as *Il21r*, *Irf1*, *Spint1*, *Slpi*, and *Ido2* possess an AHRE motif, and thus represent putative canonical AhR targets. However, further key immune genes, e.g., *Il12rb1*, *Fcgr1*, and *Cd84* were induced upon AhR activation and do not possess an AHRE motif, indicating yet unknown non-canonical and secondary mechanisms to be involved. This study gained multiple evidence that an AhR-dependent expressional induction and autocrine/paracrine signaling of type I and type II interferons may contribute to the here described modulation of Mϕ activation. However, the majority of DEGs is only significantly affected by BaP, but high correlation of whole-genome expression patterns might point to affinity and concentration effects of the used ligands. Nonetheless, it cannot be excluded that the effects are due to different binding modes of BaP (rigid aromatic 5-ring structure) and I3C (smaller, more flexible indole structure) in the AhR’s ligand binding pocket. Evidence for different binding models has been delivered by Tagliabue et al. using computational modeling, among others (61). In particular, some DEGs, i.e., *Gsta3* and *Igf1r*, possess specific or even contrary modes of regulation. Both, yet unknown non-canonical and secondary mechanisms as well as ligand-specific induction of target genes indicate, that more in-depth study is needed to completely understand the molecular mechanisms of AhR signaling in order to be prospectively utilized in immunomodulatory therapies.

## Data availability statement

The datasets presented in this study can be found in online repositories. The names of the repository/repositories and accession number(s) can be found below: GSE223122 [GEO; (82)].

## Ethics statement

The animal study was reviewed and approved by Landesdirektion Sachsen Referat 25 Braustraße 2 04107 Leipzig.

## Author contributions

JL was the principle investigator, conceptualized and designed the study, interpreted the data, drafted and revised the manuscript. JS and JH contributed equally, designed the study, performed cell culture and validation experiments, analyzed and interpreted the data, created the figures, drafted the initial manuscript, and share first authorship. SR performed cell culture and RT-qPCR experiments, CB and DL performed RNA-seq experiments, whereas KR and CK analyzed RNA-seq data. JS and KR designed the computational screen for known AHRE motifs in promoter regions. JS conducted the AHRE motif discovery, analyzed and interpreted data. SR, CB, DL, KR and CK critically reviewed and revised the manuscript. UK and SK interpreted the data and critically reviewed and revised the manuscript. All authors approved the final manuscript and agree to be accountable for all aspects of the work. All authors contributed to the article and approved the submitted version.
