# Supplementary material for: Transcriptomic signatures reveal a shift towards an anti-inflammatory gene expression profile but also the induction of type I and type II interferon signaling networks through aryl hydrocarbon receptor activation in murine macrophages
Source: Front Immunol. 2023 May 23;14:1156493. doi: 10.3389/fimmu.2023.1156493 (PMC10242070; doi:10.3389/fimmu.2023.1156493)
Supplement: Supplementary file 1 [file Image_1.pdf]

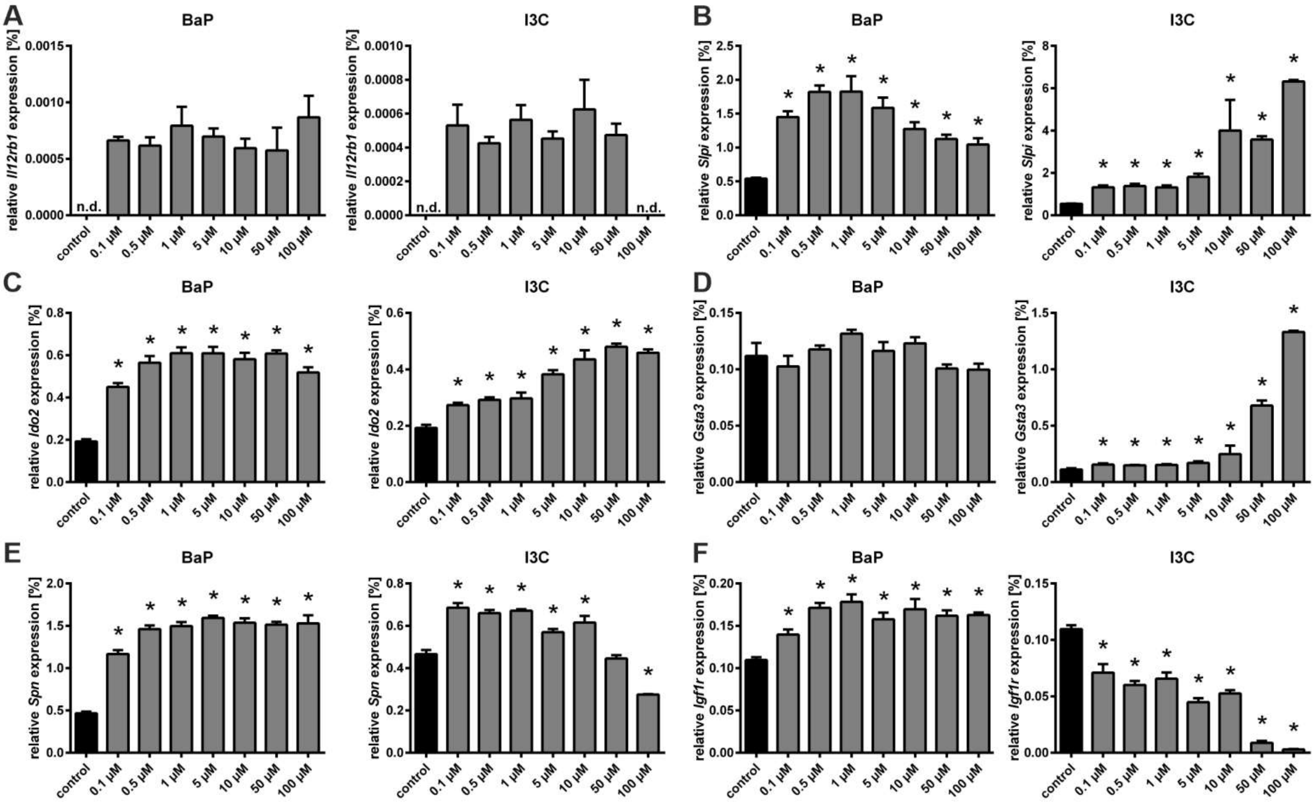

Supplementary Figure 1. BMMs from wild-type mice were treated with indicated concentrations of BaP and I3C, respectively, or DMSO as control for 6 h. Changes in gene transcription were assessed by real-time PCR after activation with hk-*S*.E. for 20 h. Relative *Il12rb1* (A), *Slpi* (B), *Ido2* (C), *Gsta3a* (D), *Spn* (E) and *Igf1r* (F) expression for BaP and I3C treatment. Data represent the mean of the relative expression ± SEM after normalization with housekeeping genes Alas1 and Hprt (n=4). * $p \leq 0.05$ indicates significant differences between treated and untreated cells.
