# Supplementary material for: Transcriptomic signatures reveal a shift towards an anti-inflammatory gene expression profile but also the induction of type I and type II interferon signaling networks through aryl hydrocarbon receptor activation in murine macrophages
Source: Front Immunol. 2023 May 23;14:1156493. doi: 10.3389/fimmu.2023.1156493 (PMC10242070; doi:10.3389/fimmu.2023.1156493)
Supplement: Supplementary file 2 [file Image_2.pdf]

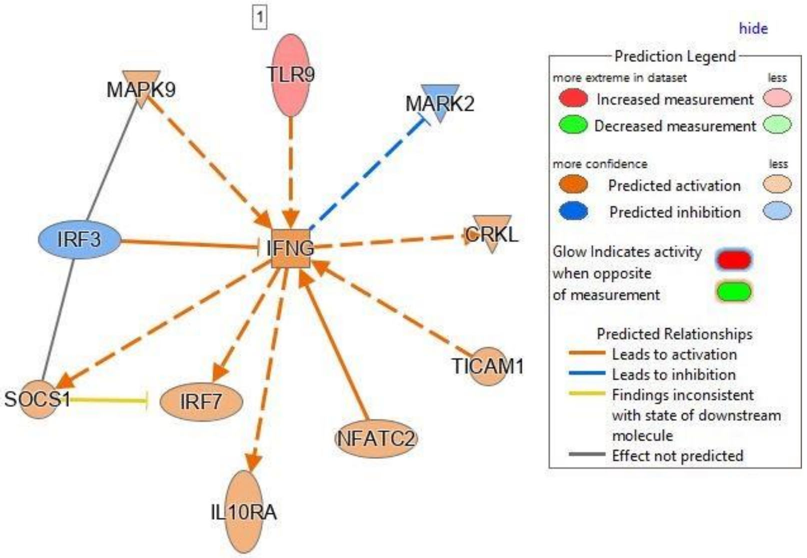

Supplementary Figure 2. Predicted upstream regulators of Ingenuity Pathway Analysis (IPA) were connected IFN-γ -centric based on IPA knowledgebase. Upstream elements were subsequently connected by Path Explorer. Activity prediction tool revealed a consistent activation of IFN-γ signalling.
