# Supplementary material for: Transcriptomic signatures reveal a shift towards an anti-inflammatory gene expression profile but also the induction of type I and type II interferon signaling networks through aryl hydrocarbon receptor activation in murine macrophages
Source: Front Immunol. 2023 May 23;14:1156493. doi: 10.3389/fimmu.2023.1156493 (PMC10242070; doi:10.3389/fimmu.2023.1156493)
Supplement: Supplementary file 5 [file Table_3.pdf]

Supplementary table 3. Statistics of correlation analyses using either all genes or only AhR dependently differential expressed genes (AhR-dep.) including Pearson's correlation coefficient (ρ), number of observations (n), calculated t and p values as well as linear regression formula and coefficient of determination (R^2).

Figure reference	correlation	genes set	ρ	n	t	p	linear regression formula	R^2
Fig. 2B (upper panel)	BaP 3h p.a. ~ BaP 20h p.a.	all	0.07	14549	8.89	$< 2.2\times10^{-16}$	$y = 0.09x + 0.01$	< 0.01
Fig. 2B (upper panel)	BaP 3h p.a. ~ BaP 20h p.a	AhR-dep.	0.44	975	15.24	$< 2.2\times10^{-16}$	$y = 0.84x + 0.06$	0.19
Fig. 2B (lower panel)	I3C 3h p.a. ~ I3C 20h p.a	all	0.34	14549	43.62	$< 2.2\times10^{-16}$	$y = 0.30x - 0.03$	0.12
Fig. 2B (lower panel)	I3C 3h p.a. ~ I3C 20h p.a	AhR-dep.	0.83	240	23.31	$< 2.2\times10^{-16}$	$y = 0.94x + 0.00$	0.70
Fig. 2C (left panel)	BaP 3h p.a ~ I3C 3h p.a.	all	0.44	14549	59.06	$< 2.2\times10^{-16}$	$y = 0.09x + 0.01$	0.19
Fig. 2C (left panel)	BaP 3h p.a ~ I3C 3h p.a.	AhR-dep.	0.79	83	11.77	$< 2.2\times10^{-16}$	$y = 1.35x - 0.90$	0.63
Fig. 2C (right panel)	BaP 20h p.a ~ I3C 20h p.a.	all	0.49	14549	68.32	$< 2.2\times10^{-16}$	$y = 0.56x - 0.04$	0.27
Fig. 2C (right panel)	BaP 20h p.a ~ I3C 20h p.a.	AhR-dep.	0.68	1070	30.21	$< 2.2\times10^{-16}$	$y = 0.69x - 0.08$	0.49
